# Effect of Successive Single-gestation Pregnancies on the Course of Maternal Human Immunodeficiency Virus Disease and Perinatal Transmission

**DOI:** 10.1155/S1064744995000135

**Published:** 1995

**Authors:** William R. Robinson, Dan Wiley, Russ Van Dyke

**Affiliations:** 1 Department of Obstetrics and Gynecology Tulane University School of Medicine Box SL 11 1430 Tulane Avenue New Orleans LA 70112 USA; 2 Department of Pediatrics Tulane University School of Medicine New Orleans LA USA; 3 Department of Medicine St. Mary's Hospital San Francisco CA USA

## Abstract

*Objective:* This study was undertaken to examine the effect of successive pregnancies over a 3-year period on the course of maternal human immunodeficiency virus (HIV) infection and the rate of perinatal transmission of HIV.

*Methods:* A retrospective analysis of 32 pregnancies in 14 known HIV-infected women vs. a matched control group of HIV-infected women who had been pregnant only once was done.

*Results:* The multiple-pregnancy group was similar to the single-pregnancy group for age, race, duration of known HIV infection, initial CD_4_ count, and date of first pregnancy. The delivery data were similar as well. The CD_4_ counts in the multiple-pregnancy group fell from 595 to 460, while counts in the single-pregnancy group fell comparably from 669 to 638, both over 37 months (*P*  = 0.1476). Five of 5 second-born infants of known serostatus vs. 8 of 21 first-born infants were HIV-infected (*P* < 0.05).

*Conclusions:* Successive pregnancies do not alter the course of HIV infection in asymptomatic women followed up to 3 years. The infants of second pregnancies of known HIV-infected women may be at higher risk for perinatal transmission.


Infectious Diseases in Obstetrics and Gynecology 2:251–254 (1995)
© 1995 Wiley-Liss, Inc.

### Effect of Successive Single-Gestation Pregnancies on the Course of Maternal Human Immunodeficiency Virus Disease and Perinatal Transmission

**William R. Robinson, Dan Wiley, and Russ Van Dyke**

*Department of Obstetrics and Gynecology (W.R.R.), Department of Pediatrics (R.V.D.), Tulane University School of Medicine, New Orleans, LA, and Department of Medicine (D.W.), St. Mary's Hospital, San Francisco, CA*

ABSTRACT

***Objective:*** **This study was undertaken to examine the effect of successive pregnancies over a 3-year period on the course of maternal human immunodeficiency virus (HIV) infection and the rate of perinatal transmission of HIV.**

***Methods:*** **A retrospective analysis of 32 pregnancies in 14 known HIV-infected women vs. a matched control group of HIV-infected women who had been pregnant only once was done.**

***Results:*** **The multiple-pregnancy group was similar to the single-pregnancy group for age, race, duration of known HIV infection, initial $CD_4$ count, and date of first pregnancy. The delivery data were similar as well. The $CD_4$ counts in the multiple-pregnancy group fell from 595 to 460, while counts in the single-pregnancy group fell comparably from 669 to 638, both over 37 months ($P$ = 0.1476). Five of 5 second-born infants of known serostatus vs. 8 of 21 first-born infants were HIV-infected ($P < 0.05$).**

***Conclusions:*** **Successive pregnancies do not alter the course of HIV infection in asymptomatic women followed up to 3 years. The infants of second pregnancies of known HIV-infected women may be at higher risk for perinatal transmission.** © 1995 Wiley-Liss, Inc.

KEY WORDS

$CD_4$, AIDS, sterilization

Women accounted for almost 15% of all new diagnoses of acquired immunodeficiency syndrome (AIDS) in the United States from June 1992 to July 1993. AIDS now represents the 5th leading cause of death in women of reproductive age.[1] In light of this problem, multiple studies of the effect of pregnancy on human immunodeficiency virus (HIV)-infected women have been reported in recent years. While most reports in this country have concluded that pregnancy has minimal impact on the progression of HIV infection,[2–6] others have suggested an association with disease acceleration as measured by $CD_4$ counts or increased rates of maternal infectious diseases.[7–10] In addition, minimal information exists concerning the effects of successive pregnancies on HIV-infected women, as most such patients have been advised to consider sterilization in light of the presumed 20–25% risk of perinatal transmission.

In communities where strong cultural or religious pressures against sterilization exist, many women opt to proceed with subsequent pregnancies after learning of their HIV infection. This report is a retrospective analysis of several parameters used to monitor disease progression, including $CD_4$ counts, opportunistic infections, and deaths in a

**Address correspondence/reprint requests to Dr. William R. Robinson, Department of Obstetrics and Gynecology, Box SL 11, Tulane University School of Medicine, 1430 Tulane Avenue, New Orleans, LA 70112.**

Received August 11, 1994

Accepted January 27, 1995

group of women who have had successive pregnancies after documentation of HIV infection vs. a case-matched group of HIV-infected women who have had only 1 pregnancy over a similar time period.

### SUBJECTS AND METHODS

Nineteen women who had been pregnant more than once since being diagnosed as HIV infected were identified from the records of 191 HIV-seropositive women at the obstetrics clinic of the Medical Center of Louisiana/Charity Hospital in New Orleans. All patients were delivered between January 1988 and October 1992. Of these, 14 had complete records available for analysis. The study patients were matched case by case with HIV-infected women who had been pregnant only once for age (for year of birth), race, date of diagnosis of HIV infection, date of delivery of first pregnancy, initial $CD_4$ count (measured during the first or second trimester), and time since diagnosis of HIV infection. No patients with AIDS-defining illnesses prior to pregnancy were included. Charts for the study and control groups were retrospectively analyzed for delivery data, $CD_4$ counts prepartum and 3–7 weeks postpartum, risk factors for HIV infection, evidence of opportunistic infections, maternal deaths, use of antiretroviral drugs, and serostatus of the child if known. The statistical analysis was performed using an analysis of variance (ANOVA) with repeated measures and Fisher's exact test as noted.

### RESULTS

As previously stated, the individuals were case matched for race (all were African American), age, and months of follow-up after first delivery. There were 32 total pregnancies in the study group (28 live births, 4 abortions). Five women in the study group used zidovudine (ZDV) during pregnancy. Three of these discontinued the drug in the first trimester due to concern about potential adverse fetal effects. One other used dideozyinosine (DDI) and one used a blinded AIDS Clinical Trials Group (ACTG) #076 study drug. In the control group, 7 women used ZDV during pregnancy, 3 of whom discontinued the drug in the first or early second trimester. One patient in the control group used a blinded ACTG #076 study drug. Three women from the study group and 4 from the control group started antiretrovirals after pregnancy was diagnosed for maternal reasons. The timing and dosage of the drugs varied widely.

**TABLE 1. Progression of $CD_4$ counts in HIV-infected women's successive pregnancies[a]**

| | Study group | Control group |
|---|---|---|
| Initial $CD_4$ count | | |
| Mean ± SE | 595 ± 75 | 669 ± 91 (NS) |
| Range | 279–175 | 228–1,327 |
| Final $CD_4$ | | |
| Mean ± SE | 460 ± 40 (NS) | 638 ± 68 |
| Range | 260–790 | 345–1,290 |

[a]All values per $mm^3$. NS = not significant.

The risk factors for HIV infection included 1 patient in the study group who had a history of intravenous (IV)-drug abuse vs. 2 in the control group. There were no patients with a history of blood or blood-product transfusion; thus, the presumed mode of transmission in the remaining patients was heterosexual intercourse.

The delivery data were compared between the study and control groups, including the rates of chorioamnionitis, endometritis, low-birth-weight infants, and type of delivery (cesarean vs. vaginal) and found to be similar as well. There was 1 case of chorioamnionitis and endometritis in both the study and control groups. Three low-birth-weight infants (<2,500 g) were born in the study group vs. 2 among the controls, and there were 5 cesarean deliveries in the study group vs. 3 among the controls.

$CD_4$ counts were also collected in both groups as listed in Table 1. The differences in $CD_4$ counts between the study and control groups were not significant at either the initial predelivery or the final postdelivery determinations ($P = 0.15$). In addition, although mean $CD_4$ counts fell in the study group from 595/$mm^3$ to 460/$mm^3$ over the 37-month period examined, the control group mean fell from 669/$mm^3$ to 638/$mm^3$ at 37 months. This difference was not significant as determined by ANOVA with repeated measures ($P = 0.1476$). The $CD_4$ percents were similar as well. Due to the wide variation in $CD_4$ counts in this relatively small analysis and the fact that $CD_4$ counts do not follow a standard distribution, the power in this analysis was low (power = 0.06).

Table 2 summarizes data on the serostatus of the infants. An infected status was documented by viral

**TABLE 2. Status of children of HIV-infected mothers from successive pregnancies**

| | Total | Deaths | HIV positive | HIV negative | Indeterminate |
|---|---|---|---|---|---|
| Study group | | | | | |
| First born | 14 | 1 | 5 | 7 | 1 |
| Second born | 14 | 1* | 4* | 0 | 9 |
| Control group | 14 | 0 | 2 | 6 | 6 |

*$P < 0.05$ (5/5 second-born HIV infected vs. 8/21 first-born of known serostatus).

cultures, polymerase chain reaction (PCR) determination, or AIDS-defining illness. Of interest, all (5/5) of the second-born children whose statuses were known were infected, including 1 child who died, vs. 8/21 first-born children ($P < 0.05$, Fisher's exact test, power = 0.65). However, in the study group, the statuses of 1 of the first-born children and 9 of the subsequent children were indeterminate. Among the controls, 6 children were of indeterminate status.

### DISCUSSION

Multiple alterations in immune status have been reported to occur in pregnancy.[11–14] Several viral illnesses other than HIV have been reported to have increased associated morbidity in pregnancy as well.[15–17] The hypothesis that HIV is similarly more aggressive in pregnancy is suggested by studies showing a fall in $CD_4$ counts, an apparent high incidence of progression to clinical illness, and a number of pregnancy-associated deaths.[7,8,18,19] These studies, however, are limited by either their lack of control groups, small numbers, or large numbers of IV-drug users with multiple concurrent problems being included in the study population. Relatively little information using a control group of nonpregnant infected women is available.

One such recent study found minimal differences in pregnant and nonpregnant women, examining both clinical and immunologic parameters.[2] A possible reason for the lack of nonpregnant control groups is that asymptomatic women at risk for infection have not consistently undergone screening for HIV until recently, in contrast to pregnant patients, who frequently have more access to testing and encouragement by health professionals.

The current study deals with this problem by utilizing a control group of infected women who had been pregnant once in comparison with women who had had successive pregnancies since learning they were infected. Due to a local cultural bias against sterilization, many HIV-infected women in this community decide to maintain their fertility. More data on the long-term effects of successive pregnancies on maternal HIV status and on the risk of perinatal transmission would allow patients to make better informed decisions regarding contraception and sterilization.

These data suggest that successive pregnancies following a diagnosis of HIV infection have minimal effect on disease progression over a 3-year follow-up period in previously asymptomatic women. No opportunistic infections or AIDS-related deaths were seen; and, although differences in $CD_4$ counts were not significant, the low power in this analysis does not rule out the possibility of a type-II error. Furthermore, since all patients had $CD_4$ counts >200, no comment can be made regarding more severely affected patients. The use of antiretroviral drugs cannot be assessed from these data. The patients using antiretroviral drugs were under the care of different physicians; therefore, the doses and timing varied. Although the use of ZDV has been shown to lessen the risk of perinatal transmission in the recently closed ACTG #076 protocol, the maternal benefits of this drug in asymptomatic patients with $CD_4$ counts >200 are uncertain.[20]

The data concerning the serostatuses of the infants suggest that second-born infants are more likely to be infected than first-born infants. Although the factors affecting perinatal transmission are poorly understood, it appears that low $CD_4$ counts may correlate with a higher risk of transmission. Whether the relatively small drop in $CD_4$ counts (595 to 460) seen in the successive pregnancy group is sufficient to account for the higher transmission rate in the second-born children is unknown. Other factors suggested as possible transmission risks such as low birth weight and chorioamnionitis were infrequent and evenly distributed among first and second births. The cesarean delivery rate was also similar (4/21 first born vs. 1/5 second born of known serostatus).

While deserving of further analysis, the increased risk seen here should be viewed with caution due to the large number of indeterminate children and relatively small sample. The decision to delay or cease childbearing would likely be profoundly affected if the children of successive pregnancies following HIV infection were shown to be at significantly higher risk for perinatal transmission.

The current study did not attempt to document the variables in prenatal care, precise timing of maternal HIV infection, or risk behaviors in the study and control groups, which may also influence transmission. Moreover, many centers including the current study site are now routinely using ZDV in pregnant women based on the information from ACTG #076. This routine will likely decrease transmission overall, making comparison of pre- and post-ZDV-use pregnancies difficult. A prospective analysis of a sufficient number of infected women at risk for successive pregnancies would be necessary to determine the precise risk of HIV transmission to subsequent children.

### ACKNOWLEDGMENTS

The authors thank Robertino Mera for his contribution to the statistical analysis in this report.

### REFERENCES

1. Centers for Disease Control: HIV/AIDS Surveillance Rep 5:2, 1993.
2. Berrebi A, Kobuch WE, Puel J, et al.: Influence of pregnancy on human immunodeficiency virus disease. Eur J Obstet Gynaecol Reprod Biol 37:211–217, 1990.
3. Alger LS, Farley JJ, Robinson BA, et al.: Interactions of human immunodeficiency virus infection and pregnancy. Obstet Gynecol 82(5):787–796, 1993.
4. Minkoff HL: Care of pregnant women infected with human immunodeficiency virus. JAMA 258:2714–2717, 1987.
5. Selwyn PA, Schoenbaum EE, Davenny K, et al.: Prospective study of human immunodeficiency virus infection and pregnancy outcome in intravenous drug users. JAMA 261:1289–1294, 1989.
6. Maynard EC, Indacochea F: Human immunodeficiency virus infection and pregnancy outcome in intravenous drug users. Am J Dis Child 14:1181–1183, 1990.
7. Robinson WR, DeShazo RD, Morgan JE: Effects of HIV infection on pregnancy. A clinical and immunologic evaluation. Ann Allergy 67:350–354, 1991.
8. Biggar RJ, Pahwa S, Minkoff HL, et al.: Immunosuppression in pregnant women infected with human immunodeficiency virus. Am J Obstet Gynecol 161:1239–1244, 1989.
9. Scott GB, Fischl MA, Klimas N, et al.: Mothers of infants with the acquired immunodeficiency syndrome. Evidence for both symptomatic and asymptomatic carriers. JAMA 253:363–366, 1985.
10. Gloeb DJ, O'Sullivan MJ, Efantis J: Human immunodeficiency virus infection in women. I. The effects of human immunodeficiency virus on pregnancy. Am J Obstet Gynecol 159:756–761, 1988.
11. Sridami V, Pacini F, Young SL, et al.: Decreased levels of helper T-cells: A possible cause of immunodeficiency in pregnancy. N Engl J Med 307:352–356, 1982.
12. Weinberg E: Pregnancy associated depression of cell-mediated immunity. Rev Infect Dis 6:814–831, 1984.
13. Birkeland SA, Kristofferson K: Lymphocyte transformation with mitogens and antigens during normal human pregnancy. A longitudinal study. Scand J Immunol 11: 321–325, 1980.
14. Cheney RT, Tomaszewski JE, Raab SJ, et al.: Subpopulations of lymphocytes in maternal peripheral blood during pregnancy. J Reprod Immunol 6:111–120, 1984.
15. Paryani SG, Arvin AM: Intrauterine infection with varicella-zoster virus after maternal varicella. N Engl J Med 314:1542–1545, 1986.
16. Stein SJ, Greenspoon JS: Rubeola during pregnancy. Obstet Gynecol 78:925–929, 1991.
17. Siegel M, Goldberg M: Incidence of poliomyelitis in pregnancy. N Engl J Med 253:841–843, 1955.
18. Minkoff HL, Nanda D, Menez R, et al.: Pregnancies resulting in infants with acquired immunodeficiency syndrome or AIDS-related complex: Follow-up of mothers, children, and subsequently born siblings. Obstet Gynecol 69:288–291, 1987.
19. Koonin LM, Ellerbrock TV, Atrash HK, et al.: Pregnancy associated deaths due to AIDS in the United States. JAMA 261:1306–1309, 1989.
20. ACTG: AIDS Clinical Trials Group Executive Summary, Abstract ACTG 076, February 20, 1994.
